# The Use of Psilocybin in the Treatment of Depressive Disorders: A Narrative Review

**DOI:** 10.7759/cureus.102694

**Published:** 2026-01-31

**Authors:** Lukasz Siwek, Marta Nowocien, Barbara Balajewicz, Angelika Samborska, Sara Szukalska, Marta Karczewska, Karolina Lichwala, Kamil Wróblewski, Paulina Wróblewska

**Affiliations:** 1 General Medicine, Szpital Miejski w Zabrzu, Zabrze, POL; 2 Faculty of Medical Sciences in Katowice, Medical University of Silesia, Katowice, POL; 3 General Medicine, Medical University of Silesia, Katowice, POL; 4 Orthopaedics and Traumatology, Dr. Tytus Chałubiński Specialist Hospital in Radom, Radom, POL; 5 General Medicine, Poznan University of Medical Sciences, Poznan, POL; 6 General Medicine, University Clinical Hospital in Poznan, Poznan University of Medical Sciences, Poznan, POL

**Keywords:** antidepressants, anxiety disorders, depression, psilocybin, psychedelics

## Abstract

Psilocybin is a psychoactive chemical compound that exerts its effects through the activation of serotonergic receptors. It occurs naturally in mushrooms of the genus Psilocybe. Despite its potential medical applications, this substance is regarded as a drug with no recognized medical use. Depression constitutes a psychiatric disorder of substantial global burden, affecting millions of individuals worldwide, with epidemiological data indicating a continuing upward trend in its prevalence. It is a complex disease entity that, despite years of research, remains not fully understood and constitutes a significant therapeutic challenge. Its pathogenesis is based on the interaction of biological, environmental, and social factors. It is estimated that by the year 2030, depression will become the leading cause of disability. The concern associated with this projection, together with human curiosity, has formed the foundation of numerous scientific studies conducted in recent years, aimed at identifying a breakthrough therapeutic approach that would expand the range of treatment options available to psychiatrists. The aim of this paper is to present the most recent reports on attempts to use the controversial substance psilocybin in the treatment of depression. Owing to promising research results demonstrating high therapeutic efficacy in comparison with conventional, currently recommended treatments, psilocybin-assisted therapy offers hope for the development of a modern therapeutic approach that provides the expected clinical outcomes, with a proven and more sustained therapeutic effect in treated patients, as well as a minimal number or complete absence of adverse effects.

## Introduction and background

Psilocybin (4-phosphoryloxy-N,N-dimethyltryptamine) is a psychodysleptic agent, a chemical compound with strong psychoactive properties that influences mood and the perception of reality [1,2]. It is a substance that has been used by humanity for thousands of years and was well known to the Maya civilization, which employed it during rituals intended to induce visions and spiritual experiences. Psilocybin was isolated and identified between the years 1955 and 1958 by Albert Hofmann in the laboratories of the Sandoz company in Switzerland [3]. It was popularized by Robert Gordon Wasson, an amateur mycologist, who in the year 1957 described his experiences with the ingestion of psilocybin-containing mushrooms during a Mazatec ritual in Oaxaca, Mexico, in a photographic essay entitled “Seeking the Magic Mushroom”, published in Life magazine [4]. 

Depression affects approximately 3.8% of the global population, including 5% of the adult population [5]. According to reports from the World Health Organization (WHO), the disorder is characterized by persistent sadness, a diminished ability to experience pleasure from previously satisfying activities, and, in some cases, anhedonia. These states may be present in affected individuals nearly every day for a minimum duration of two weeks [5]. Depression affects nearly all aspects of a patient’s life. It may lead to impairments in concentration, productivity, and the ability to function in society, difficulties in maintaining social relationships, and decreased appetite and a reduced sense of vital energy. Suicidal ideation occurs in approximately 60 percent of patients, while the actual risk of attempting suicide among individuals with depression is estimated at 15 to 20 percent. According to data from the WHO, approximately 700, 000 people die by suicide each year [5]. 

In patients suffering from depression, neuroimaging studies have revealed abnormalities in brain function. A reduction in gray matter volume and alterations in the activity of brain regions involved in emotion processing have been observed in comparison to healthy individuals. These differences are primarily noted in the amygdala, insula, and the temporal and prefrontal cortices [6]. Additionally, effects on cortico-limbic activity have been demonstrated [7]. Due to the heterogeneity of changes observed in diagnosed patients, it is challenging to precisely determine the alterations that occur in the human brain during the progression of the disorder. Similarly, the etiology of depression is not yet fully understood; it is recognized that the onset of the disorder is influenced by genetic, epigenetic, environmental, and individual factors related to a person’s life experiences [8]. Confirmed risk factors include female sex - women are estimated to be affected twice as frequently as men, comorbid medical conditions, including Alzheimer’s disease Parkinson’s disease, Huntington’s disease, and thyroid disorders, sleep disturbances, experienced trauma, substance abuse, addiction to alcohol or drugs, low socioeconomic status, pregnancy, the puerperium, and the postpartum period: the prevalence of depression during pregnancy is estimated at 12.7%, whereas during the postpartum period, it ranges from 1% to 5.7% [9]. 

In current treatment methods for depressive disorders, despite significant progress compared to previous decades, many patients still do not achieve full remission of the disease and experience numerous adverse effects. One of the innovative and still investigational therapies for depression involves the use of a substance found in psychoactive mushrooms. Therefore, the objective of this narrative review is to assess the use of psilocybin in the treatment of depressive disorders.

## Review

Depression diagnosis 

Accurate diagnosis of depression is crucial for improving a patient’s health status. Clinical interviews and conversations constitute the primary sources of information; however, it is also necessary to exclude other medical conditions that may present with similar symptoms. During the diagnostic process, specialists may utilize a variety of tools to assist in assessing the severity of depression. These include, among others, the following.

PHQ-9 (Patient Health Questionnaire)

This is a nine-level questionnaire in which the patient responds to questions regarding, among other aspects, their mood, thoughts, and concentration, assigning a score from 0 to 3 for each item. The scores are then summed and interpreted by a specialist. A score of 5-9 indicates mild depression, 10-14 moderate depression, 15-19 moderately severe depression, and 20 or higher severe depression [10]. The PHQ-9 scale is considered reliable and valid for depression screening [11]. 

QIDS (Quick Inventory of Depressive Symptomatology)

This is a 16-level assessment that can be divided into QIDS-SR (Quick Inventory of Depressive Symptomatology - Self Report) and QIDS-C (Quick Inventory of Depressive Symptomatology - Clinician Rating) [12]. 

HAM-D (Hamilton Depression Rating Scale)

This is a 21-point clinician-administered scale used to determine the severity of depressive symptoms [13]. 

GRID-HAMD-17 (GRID-Hamilton Depression Rating Scale)

This is an enhanced version of the HAM-D, incorporating additional features while maintaining high efficacy [14]. 

HAM-A (Hamilton Anxiety Rating Scale)

This is a 14-level scale assessing both psychological anxiety and somatic tension. Evaluation using this scale is conducted by an experienced clinician or researcher; it is not intended for self-assessment [13]. 

*STAI (State-Trait Anxiety Inventory)* 

This is a 20-level self-report scale developed by Charles Spielberger, consisting of two components that distinguish anxiety as a temporary emotional state (STAI-S, State Anxiety Scale) from anxiety as a personality trait (STAI-T, Trait Anxiety Scale) [15]. 

The Profile of Mood States (POMS)

This is a self-report questionnaire designed to assess transient emotional states and mood. The full version consists of 65 items, organized into six subscales: Tension‑Anxiety, Depression‑Dejection, Anger‑Hostility, Vigor‑Activity, Fatigue‑Inertia, and Confusion‑Bewilderment. Respondents rate the extent to which they have experienced each emotion using a five-point Likert scale. Scores for each subscale, as well as the Total Mood Disturbance index, provide an overall measure of negative mood intensity and level of energy. POMS is widely used in clinical and psychological research to monitor changes in mood [16]. 

HADS (Hospital Anxiety and Depression Scale)

This is a 14-level scale designed to assess the frequently co-occurring symptoms of anxiety and depression. It focuses on non-physical symptoms, making it suitable for use by individuals with certain levels of disability. The scale is divided into subscales, with seven items assessing anxiety and seven assessing depression. The subscales include HADS-T (HADS-Total Score), HADS-A (HADS-Anxiety), and HADS-D (HADS-Depression) [17]. 

*MADRS (Montgomery-Åsberg Depression Rating Scale)* 

This is a scale used to evaluate the severity of depression, commonly employed to assess treatment efficacy in antidepressant therapy [18]. 

*BDI (Beck Depression Inventory)* 

This is a 21-point questionnaire in which the patient selects one of four statements for each item that best describes their mood. The scores are then summed, with higher total scores indicating greater severity of depressive symptoms [19]. 

CES-D (Center for Epidemiologic Studies Depression Scale)

This is a 21-point test in which patient scores range from 0 to 60 points. The total score reflects the frequency of depressive symptoms [20]. 

MGH ATRQ (Massachusetts General Hospital Antidepressant Treatment Response Questionnaire)

This is a self-report scale used to determine treatment resistance in major depressive disorder. It defines six weeks of receiving an adequate dose of an antidepressant as an appropriate treatment duration. Additionally, it provides detailed criteria regarding proper dosing for each of the most commonly used antidepressant medications [21]. 

Depression: contemporary treatment methods 

The treatment of depression depends on the severity of the disorder and the number of depressive episodes experienced. Current guidelines for the treatment of depression emphasize the importance of an integrated therapeutic approach that includes psychotherapy, psychoeducation, and pharmacological treatment; additionally, in selected clinical cases, particularly in severe and treatment-resistant forms of depression, electroconvulsive therapy is also recommended [5,22]. 

Psychoeducation 

Effective treatment of depression relies heavily on the active involvement of both the patient and their support system, including family members, friends, and caregivers. Patients should receive comprehensive information about their condition, available therapeutic options, and strategies for maintaining mental well-being. They are encouraged to take an active role in choosing the most suitable treatment approach. When pharmacological therapy is selected, patients should be thoroughly informed about its potential benefits and risks. Moreover, they should be educated on the correct use of medications, the importance of consistent adherence, and the need to avoid skipping doses [23]. 

Psychotherapy 

Psychotherapy is considered an integral component of depression treatment, although its role depends on the severity of the disorder. In cases of moderate to severe depression, pharmacotherapy is typically the treatment of choice, whereas for mild depressive episodes, psychotherapy alone may be sufficient. Numerous psychotherapeutic approaches are employed in the management of depression, including cognitive-behavioral therapy, behavioral activation, and interpersonal psychotherapy [23]. 

The benefits of psychotherapy are substantial; however, it is important to acknowledge its limitations. Psychotherapy typically requires several months of active collaboration between the patient and a specialist, involves certain financial costs, and is labor-intensive, demanding significant commitment and consistency from the patient [24]. 

*Pharmacological Treatment* 

Proper selection of pharmacotherapy tailored to the individual needs of the patient is crucial in the management of depression. Factors to consider include current medications, dietary supplements, comorbid conditions, lifestyle, age, and numerous other variables that may influence treatment outcomes. The anticipated therapeutic effect of an antidepressant should become noticeable within two to four weeks. If no clinical improvement is observed after six to eight weeks of correctly administered treatment at the maximum tolerated dose, a trial with an alternative pharmacological agent should be initiated [23]. 

First-line antidepressant medications currently used include the following.

SSRIs (selective serotonin reuptake inhibitors): The drugs in this class act by increasing serotonin (5-hydroxytryptamine; 5-HT) levels in the body. By inhibiting the serotonin transporter (SERT), they prevent the selective reuptake of serotonin into the cytoplasm of nerve cells, resulting in elevated extracellular serotonin concentrations and increased availability in the synaptic cleft [24]. Due to their minimal effects on dopamine, norepinephrine, histamine, and acetylcholine, SSRIs are considered relatively safe. However, potential adverse effects include sexual dysfunction, gastrointestinal disturbances, sleep disorders, QT interval prolongation, dizziness, and headaches. They may also cause anxiety and increase the risk of suicidal thoughts, particularly in children and adults under the age of 25 [25]. Another possible adverse effect is serotonin syndrome, a rapidly developing condition characterized by autonomic hyperactivity, changes in mental status, tachycardia, hypertension, hyperthermia, nausea, vomiting, restlessness, and disorientation [25,26]. The most commonly prescribed SSRIs include fluoxetine, sertraline, citalopram, and escitalopram. 

SNRIs (serotonin-norepinephrine reuptake inhibitors): The mechanism of action of these drugs involves modulation of serotonergic and noradrenergic neurotransmission through inhibition of the SERT and the norepinephrine transporter (NET) [27]. The most commonly prescribed medications in this class include venlafaxine and duloxetine. The efficacy of SSRIs and SNRIs has been shown to be very similar. Studies report that 40-60 out of 100 patients receiving these medications achieve a therapeutic response, compared to 20-40 out of 100 individuals in placebo groups [28]. 

SARI (serotonin antagonist and reuptake inhibitor): A representative of the SARI class is trazodone, which acts by blocking 5-HT₂A, 5-HT₂C, and α₁-adrenergic receptors, and also functions as a weak serotonin reuptake inhibitor [26]. Potential adverse effects of trazodone include headaches, dizziness, fatigue, drowsiness, and, in some cases, visual hallucinations. Prior to initiating treatment and during therapy, regular monitoring is recommended, as the drug is metabolized in the liver and kidneys and may exert toxic effects on these organs [29]. 

NDRIs (norepinephrine-dopamine reuptake inhibitors): Bupropion is an example of a medication in the NDRI class and also acts as a competitive inhibitor of CYP2D6. Unlike the previously mentioned drug classes, it does not affect serotonin reuptake but inhibits the reuptake of norepinephrine and dopamine [30]. The most serious adverse effect associated with bupropion use is the risk of seizures [27]. 

NASSAs (noradrenergic and specific serotonergic antidepressants): Mirtazapine exhibits serotonergic activity that is not directly related to the blockade of serotonin or noradrenergic receptors. Through 5-HT₁A receptors, it inhibits central α₂-adrenergic autoreceptors and heteroreceptors, while postsynaptically blocking 5-HT₂ and 5-HT₃ receptors [23]. It also acts on H₁ histamine receptors, which can induce sedative effects [27]. 

RIMAs (reversible inhibitors of monoamine oxidase A) and MAOIs (monoamine oxidase inhibitors): RIMAs are reversible inhibitors of monoamine oxidase A, whereas MAOIs are a broader class of drugs that inhibit monoamine oxidase enzymes, affecting the metabolism of neurotransmitters such as serotonin, norepinephrine, and dopamine [8]. 

Second-line medications: TCAs (tricyclic antidepressants, also referred to as tricyclic low potency drugs (TLPDs)) are classified as second-line treatments for depression due to their numerous adverse effects, despite their high therapeutic efficacy [23]. 

NRIs (norepinephrine reuptake inhibitors): NRIs enhance adrenergic neurotransmission through selective inhibition of the NET, which consequently increases dopamine and norepinephrine (NE) levels in the prefrontal cortex. Adverse effects are primarily associated with elevated NE and include constipation, dry mouth, urinary disturbances, insomnia, anxiety, agitation, impotence, and dose-dependent hypotension [27]. Reboxetine is a representative drug of this class.

Treatment efficacy 

Although multiple treatment options exist, including combinations of medications from different classes and various psychotherapeutic approaches, none provide a completely effective solution. Current therapies are constrained by numerous potential side effects and limited accessibility. According to the WHO, more than 75 percent of individuals with mental disorders in low- and middle-income countries do not receive adequate care [5]. Conventional antidepressant treatments fail to produce improvement in approximately 30 percent of patients [8], and it is estimated that between one-third and one-half of individuals with diagnosed major depressive disorder (MDD) either do not respond to treatment or achieve only partial relief of symptoms. Patients who do not respond after two trials with standard antidepressants may be classified as having treatment-resistant depression [31].

Psilocybin in the treatment of depression 

The evaluation of psilocybin’s efficacy in the treatment of depressive disorders is based on analyses of independent studies conducted in recent years. These investigations build upon research from the 1950s to 1970s, which explored the use of hallucinogenic substances in addressing anxiety, despair, and social isolation. Such studies encourage a renewed consideration of the medical potential of these compounds under controlled hospital conditions.

The first scientific publication cited herein as an example is a study published in 2011 [32]. This study represented a pivotal advancement in the exploration of psilocybin as a therapeutic intervention for individuals with reactive anxiety, constituting the first study of its kind conducted after an interruption exceeding 35 years.

The study group consisted of 12 carefully selected individuals with advanced-stage cancer and a diagnosed acute stress, anxiety, or adjustment disorder according to DSM-IV criteria. Eleven of the 12 participants were women aged between 36 and 58 years. Four volunteers had never previously been exposed to hallucinogens, another four had such experience over 30 years prior to the study, two had used hallucinogens more than five years before participation, and the remaining two had taken a hallucinogen within the year preceding the study. 

After appropriate preparation, each patient underwent two experimental therapeutic sessions spaced several weeks apart. In one session, the patient received active psilocybin at a dose of 0.2 mg/kg, while in the other session, they received a placebo, consisting of niacin at a dose of 250 mg. Both substances were administered in clear corn-starch capsules accompanied by 100 ml of water. 

Each session lasted six hours, during which members of the therapeutic team remained at the patient’s bedside. The specific treatment administered in a given week was known only to the study pharmacist. Sessions concluded with a discussion and the completion of assessment instruments, including various inventories and self-report questionnaires, which were administered from two weeks prior to the study through six months after its conclusion. Additionally, the therapeutic team maintained contact with the participants throughout the six-month follow-up period. 

To evaluate the study outcomes, psychological assessments were conducted the day before each experimental session using the BDI, POMS, and STAI scales. At the conclusion of each session, participants completed the five-dimensional Altered States of Consciousness Profile (5D-ASC), the Brief Psychiatric Rating Scale, as well as the POMS and STAI. On the day following the session, BDI, POMS, and STAI assessments were repeated. Final BDI, POMS, and STAI measurements were completed by patients two weeks after each session and then monthly throughout the subsequent six-month follow-up period. 

Despite the administration of a low dose of psilocybin, therapeutic benefits were observed in quantitative psychological assessments. The POMS did not show statistically significant improvement, although there was a trend toward positive outcomes. The STAI revealed no significant changes up to two weeks post-treatment; however, the six-month follow-up indicated a sustained reduction in anxiety, stress, and tension.

BDI scores also showed improvement. The results indicated no noticeable change from one day before capsule administration to two weeks post-treatment in the placebo condition. In contrast, psilocybin therapy demonstrated a trend toward decreased BDI scores, reaching nearly a 30% reduction when comparing one month after the second session to the baseline prior to the first session. This difference persisted and remained significant throughout the six-month follow-up assessments. 

Additionally, blood pressure (BP) and heart rate (HR) were measured 30 minutes prior to substance administration, immediately before administration, and at 60-minute intervals over the subsequent six hours. Following psilocybin administration, peak HR was observed two hours post-dose, with a mean of 81.5 beats per minute (SEM), which was statistically significant compared to 70.4 beats per minute during the placebo session.

Similarly, BP reached its peak two hours after psilocybin ingestion, with a mean value of 138.9 mmHg, compared to 117.0 mmHg during the placebo session. The mean diastolic BP (SEM) during psilocybin sessions was 75.9 (3.4) mmHg, whereas during placebo sessions it was 69.6 (2.7) mmHg.

The findings, together with the lack of negative effects, including psychological side effects, indicate that controlled psilocybin administration may serve as an effective alternative to traditional therapies for managing anxiety associated with advanced-stage cancer [32].

Another significant study was published in 2016 [33]. This study was built upon the research conducted at Harbor-UCLA (Grob et al., 2011) and represented a further step in investigating the effects of psilocybin treatment for depression. In this trial, the study group was considerably larger, comprising 18 women and 11 men aged 22 to 75 years. All participants had cancers of various types and locations, with 62% classified as stage III or IV. Adaptive disorders were observed in 90% of participants, while 10% presented with anxiety disorders. None of the participants were receiving psychotropic medications at the time of enrollment. 

After appropriate preparation, each participant underwent a double-blind, controlled trial in which they received a single dose of either 0.3 mg/kg psilocybin or niacin, combined with psychotherapy. A seven-week washout period was implemented between administrations of the two substances, during which participants were continuously observed, assessed, and evaluated. The total duration of the study was approximately nine months. 

The medical safety profile of psilocybin, including its effects on cardiovascular parameters, was consistent with findings reported in earlier studies, with no serious or clinically significant adverse events attributable to the administration of the substance observed throughout the trial.

The efficacy of depression treatment was assessed using HADS-T, HADS-A, HADS-D, BDI, STAI-S, and STAI-T scores. These measures demonstrated significant differences between the experimental and control groups prior to the administration of the second dose. Unlike niacin, psilocybin administration produced an immediate and substantial reduction in both anxiety and depressive symptoms, which persisted up to seven weeks post-administration. For example, seven weeks after the first psilocybin dose, 83% of participants met the criteria for an antidepressant response (BDI), and 58% met the criteria for an anxiolytic response (HADS-A). Follow-up over 6.5 months confirmed sustained antidepressant or anxiolytic effects in 60-80% of participants [33]. 

The next study whose findings are discussed in the present review is a randomized, double-blind, crossover study that was conducted without administering niacin to participants [34]. One arm of the trial investigated the effects of a very low dose of psilocybin, comparable to a placebo (1 or 3 mg/70 kg), while the other arm examined the effects of a high dose of psilocybin (22 or 30 mg/70 kg). 

Fifty-six participants were selected from a pool of 566 volunteers, of whom 51 completed at least one session. The gender distribution was nearly equal. All participants had a diagnosis of potentially life-threatening cancer. As in previous studies, cancer types and locations were diverse, with 65% of participants experiencing either recurrence or metastasis. Additionally, participants presented with chronic adjustment disorders with anxiety (11 participants) or mixed anxiety and depressed mood (11), dysthymic disorder (5), generalized anxiety disorder (GAD) (5), MDD (14), or comorbid diagnoses including GAD and MDD (4) or GAD and dysthymic disorder (1). 

Therapeutic sessions began once participants were assigned to either the experimental or control group. Two sessions compared the effects of extremely different psilocybin doses, with a five-week interval between sessions. Strict adherence to instructions for both participants and observers minimized expectancy effects, which play a significant role in the qualitative outcomes of psilocybin administration. 

No lasting adverse effects were observed during the study. Medical interventions were not required, and any cardiovascular events that occurred were mild and transient. The primary measures of clinically significant response and remission-GRID-HAMD-17 for depressive symptoms and HAM-A for anxiety-demonstrated substantial therapeutic effects of psilocybin, which persisted for up to six months. Five weeks after the first session, 92% of participants in the high-dose group exhibited a clinically significant response (≥50% reduction from baseline) on the GRID-HAMD-17, compared to 32% in the low-dose group. For the HAM-A, 76% of the high-dose group achieved a significant response at five weeks post-session one, whereas only 26% of the low-dose group met this criterion. 

Remission of symptoms, as measured by the GRID-HAMD-17, persisted in an average of 65% of participants even six months after the final session, while HAM-A assessments showed sustained remission in 56.5% of participants. For other measures, including GRID-HAMD, BDI, and HADS-D, the high-dose group achieved faster and greater therapeutic effects compared to the low-dose group, with benefits maintained for at least six months. The overall clinical response rate at six months for depression and anxiety was 78% and 83%, respectively [34]. 

In the same year, another noteworthy study was published [35]. In this study, unlike previous trials, participants were individuals with diagnosed moderate to severe, treatment-resistant, unipolar depression, without any oncological history. The study also differed in the absence of a control group. The experimental cohort consisted of 12 participants, with an equal distribution of women and men. Inclusion criteria required a HAM-D score of 17 or higher and a lack of improvement despite at least six weeks of prior conventional treatment. Nine of the twelve patients suffered from severe or very severe depression (BDI ≥ 30). Exclusion criteria included a personal or immediate family history of psychotic disorders, current substance dependence, or a positive pregnancy test. 

Following successful preparatory procedures, which included a 60-minute comprehensive session with an assigned psychiatrist for each participant, patients were enrolled in two experimental sessions separated by a seven-day interval. Both sessions were conducted in comfortable and safe settings. During the first session, participants received two capsules containing a total of 5 mg of psilocybin, and in the second session, one week later, the dose was increased to 25 mg. The study group tolerated psilocybin well and experienced no serious or unexpected adverse events. 

Baseline QIDS scores showed a significant reduction following psilocybin administration, with the maximum effect observed at two weeks (p = 0.002) and a sustained, statistically significant effect lasting at least three months. According to BDI criteria, eight of the 12 participants achieved full remission of depressive symptoms after one week. At three months, seven participants maintained a satisfactory response (≥50% reduction in BDI score from baseline), and five participants (42%) continued to exhibit complete remission of symptoms. Additionally, STAI-T anxiety scores and SHAPS anhedonia scores demonstrated substantial improvement [34].

The largest study among those cited to date was published in 2022 [36]. This study was conducted across 22 centers located in 10 countries: in Europe (Czech Republic, Denmark, Germany, Ireland, the Netherlands, Portugal, Spain, and the United Kingdom) and North America (Canada and the United States). A team of well-trained investigators and therapists carried out the trial on 233 enrolled participants, with the primary aim of evaluating the efficacy of psilocybin-assisted therapy for treatment-resistant depression. 

The mean age of participants was 39.8 years, with 52% being women. All met DSM-V criteria for single or recurrent episodes of treatment-resistant depression without psychotic features, as confirmed through clinical evaluation and documentation consistent with the Mini-International Neuropsychiatric Interview. Prior to psilocybin therapy, according to MADRS scores, 30% of participants had moderate depression (MADRS: 20-30) and 68% had severe depression (MADRS: ≥31); 71% had moderate HAM-D scores, while 29% had severe scores. Additionally, all participants had failed to respond to 2-4 prior conventional treatment attempts, as assessed using the MGH ATRQ. 

Participants were randomly assigned to three groups: 79 individuals (mean MADRS = 31.9) in the first group, 75 (mean MADRS = 33) in the second group, and 79 (mean MADRS = 32.7) in the third group. The study was conducted as a double-blind trial, with participants receiving 25 mg (group 1), 10 mg (group 2), or 1 mg of psilocybin (group 3). Following the session, a 12-week follow-up period was implemented. Participants were assessed by clinicians using the MADRS on the second day post-session and at weeks 1, 3, 6, 9, and 12. 

Adverse events were reported in 66 participants: 84% in the 25 mg group, 75% in the 10 mg group, and 72% in the 1 mg group. In addition to headaches, nausea, and fatigue, the 25 mg and 10 mg psilocybin groups exhibited a relatively higher incidence of suicidal ideation or self-harming behavior.

At week 3, response to treatment was observed in 37% of participants receiving 25 mg of psilocybin, compared with 19% in the 10 mg group and 18% in the 1 mg group; corresponding remission rates were 29%, 9%, and 8%, respectively. The mean reduction in Montgomery-Åsberg Depression Rating Scale (MADRS) scores from baseline to week 3 amounted to −12 points in the 25 mg group, −7.9 points in the 10 mg group, and −5.4 points in the 1 mg group, demonstrating the superior efficacy of the highest dose. A between-group comparison revealed a 6.6-point greater reduction in mean MADRS score in the 25 mg group relative to the 1 mg group, whereas no statistically significant difference was identified between the 10 mg and 1 mg doses. Notably, a subset of participants in both the 1 mg and 10 mg groups achieved a reduction of at least 50% in MADRS scores compared with baseline.

The sustained response rate at week 12 was 20% in the 25 mg group, 5% in the 10 mg group, and 10% in the 1 mg group. However, due to the failure of hierarchical testing, definitive conclusions cannot be drawn [36].

Finally, a study published in 2023 is discussed [37]. The most recent among the cited studies employed a double-blind design, with a single dose of psilocybin (25 mg) and niacin (100 mg) as a placebo. The trial included 104 adults aged 21-65 years with moderate to severe depressive disorder. Over the 43-day study period, psilocybin administration resulted in a clinically significant improvement in depressive symptoms, as measured by MADRS, with an average reduction of 12.3 points favoring psilocybin treatment. Consistent with previous studies, no serious adverse events were reported [37]. 

Discussion 

Existing studies are promising, demonstrating a positive therapeutic effect of psilocybin in patients with depressive disorders. However, these studies have notable limitations, including small sample sizes, the exclusion of participants taking psychotropic medications due to potential interactions with psilocybin, and challenges in effectively blinding placebo groups. Additionally, objective assessment of treatment outcomes remains limited. 

Legal status presents another significant barrier to psilocybin therapy. In most countries, psilocybin is classified as an illegal substance with no recognized medical use and a high potential for abuse. Implementing such therapy would require decriminalization for medical purposes, accompanied by a comprehensive economic evaluation conducted by health economists in collaboration with scientists [38]. Although substantial interdisciplinary effort would be necessary, the potential benefits for patients could outweigh these challenges, particularly since prior studies included participants who had not responded to conventional treatments, yet demonstrated improvement following psilocybin therapy. 

One advantage of psilocybin therapy is its low incidence of adverse effects. In the studies conducted to date, negative outcomes were generally mild and included transient nausea, temporary headaches, psychological discomfort, visual distortions, and minor cardiovascular events that did not result in lasting or clinically significant complications. Observed elevations in BP and HR resolved spontaneously without medical intervention [39]. 

Comparing the efficacy of psilocybin with standard antidepressant medications such as escitalopram and fluvoxamine, psilocybin produces a rapid and clinically significant reduction in depressive symptoms following one or a few sessions supported by psychotherapy, with effects persisting for several weeks [33]. In contrast, escitalopram and fluvoxamine exert their antidepressant effects gradually over several weeks of daily administration, with documented efficacy in standard pharmacotherapy protocols for depression [40,41]. These differences highlight distinct temporal profiles between the interventions, with psilocybin potentially providing faster symptom relief, whereas SSRIs require consistent, ongoing administration to achieve full therapeutic effects.

## Conclusions

Clinical studies indicate that psilocybin produces a reduction in depressive symptoms in individuals with major depressive episodes and treatment-resistant depression. Randomized and placebo-controlled trials have shown that a single dose of psilocybin, administered in the context of psychotherapeutic support, results in a substantial decrease in depression rating scale scores, including QIDS-C, MADRS, and PHQ-9, with effects persisting for several weeks post-administration. Pilot studies in patients with treatment-resistant depression further demonstrated improvements not only in depressive symptom severity but also in measures of emotional functioning and quality of life. Neurobiological research indicates that the reduction in depressive symptoms is associated with modulation of neural networks involved in mood regulation and processing of negative emotions, including cortical and subcortical regions. Pharmacological analyses confirm that psilocybin acts through interactions with serotonin receptors, which are linked to the observed alleviation of depressive symptoms. Based on current evidence, psilocybin therapy in the treatment of depression could represent a viable alternative to conventional approaches for many patients. Therefore, it is essential to continue advancing research and exploring innovative therapeutic strategies, among which psilocybin-assisted therapy may hold significant potential. 
